# A Novel Digital Patient-Reported Outcome Platform (Noona) for Clinical Use in Patients With Cancer: Pilot Study Assessing Suitability

**DOI:** 10.2196/16156

**Published:** 2021-05-06

**Authors:** Maria Kristiina Peltola, Paula Poikonen-Saksela, Johanna Mattson, Timo Parkkari

**Affiliations:** 1 Department of Oncology Helsinki University Hospital Comprehensive Cancer Center Helsinki University Helsinki Finland; 2 Clinius ltd Helsinki Finland

**Keywords:** electronic patient-reported outcome, adverse events, patients with cancer

## Abstract

**Background:**

As the incidence of cancer is on the rise, there is a need to develop modern communication tools between patients and the medical personnel. Electronic patient-reported outcome (ePRO) measures increase the safety of cancer treatments and may have an impact on treatment outcome as well. ePRO may also provide a cost-efficient way to organize follow-up for patients with cancer. Noona is an internet-based system for patients to self-report symptoms and adverse events of cancer treatments from home via a computer or a smart device (eg, smartphone, tablet).

**Objective:**

In this pilot study, we assessed the suitability of a novel ePRO application (Noona) for patients with cancer, nurses, and doctors at the Helsinki University Hospital, Finland.

**Methods:**

The study included 44 patients with cancer (different solid tumor types) and 17 health care professionals (nurses or medical doctors). Patients were either operated or received systemic treatment or radiotherapy. Patients reported their symptoms to the medical staff via Noona. In addition, patients and clinicians answered a questionnaire, based on which Noona’s suitability for clinical use was evaluated in terms of usability (ease of use, operability, and learnability), reliability (subjective opinion of the participant), and incidence of harmful events reported by the participants.

**Results:**

A total of 41/44 (93%) patients and 15/17 (88%) professionals reported that the program was easy or quite easy to use; 38/44 (86%) patients and 11/17 (65%) professionals found Noona reliable, and 38/44 (86%) patients and 10/17 (59%) professionals would recommend Noona to other patients or their colleagues. No harmful incidences caused by the use of Noona were reported by the patients; however, 1 harmful incidence was reported by one of the professionals.

**Conclusions:**

The majority of the participants felt that Noona appeared reliable and it was easy to use. Noona seems to be a useful tool for monitoring patient’s symptoms during cancer therapy. Future studies will determine the impact of this ePRO platform in routine clinical practice.

## Introduction

According to the Finnish Cancer Registry, approximately 30,000 people receive cancer diagnoses in Finland every year, and the number of patients with cancer continues to rise.

Cancer treatments cause adverse events and long-term consequences. Patients receiving radiotherapy may experience irritation, redness of skin, pain, and fatigue. If the acute reactions remain improperly treated, patients may discontinue radiotherapy, leading to a loss of local control [1].

During chemotherapy patients commonly experience side effects such as nausea, fatigue, neutropenic infections, mucositis, peripheral neuropathy, and pain. These side effects impair patients’ quality of life and may require emergency room visits, hospital stays, reductions in the following chemotherapy doses, or lead to treatment interruption [2]. Recognizing adverse events of chemotherapy early remains important to ensure proper medical interventions [3].

Digital communication between patients and cancer clinics via electronic patient-reported outcomes (ePROs) enables early detection of adverse events during chemotherapy, decreases emergency room visits and hospitalization, increases quality of life, and may even improve survival [3]. In the study by Denis et al [4], after primary treatment of lung cancer, disease relapse was detected earlier and the patients lived longer if they reported their symptoms via electronic software, compared with traditional follow-up visits. The overall survival was 19 months (95% CI 12.5 to noncalculable) in the study arm, compared with 12 months (95% CI 8.6-16.4, *P*=.001) in the control arm [4]. Because of these advantages, ePROs will likely be implemented in routine cancer care in the near future [5].

The aim of this study is to describe the usability of the first version of Noona, a web-mediated PRO application.

## Methods

The investigated ePRO tool Noona is a web-mediated application developed by Noona Healthcare Oy in collaboration with Helsinki University Hospital Comprehensive Cancer Center. The Noona mobile service is developed for remote monitoring of patients with cancer, and to be used as a support tool for communication between patients with cancer and health care professionals. Noona is an online application, which can be used with a web browser and a suitable device, such as desktop, laptop, tablet, and smartphone. Noona has 2 clear user groups: patients with cancer and cancer care professionals, specifically nurses and doctors working in cancer hospitals. At the beginning of their treatment, patients with cancer are registered to Noona, and they will continue to use Noona during the follow-up and rehabilitation periods. During the treatment phase, Noona is designed to evaluate symptoms and recovery progress based on patient-reported outcome data. During the follow-up and rehabilitation periods, the intended use is to enable fluent and accessible communication between the patient and the professionals, and to monitor patient recovery from cancer and related symptoms.

**Figure 1:**
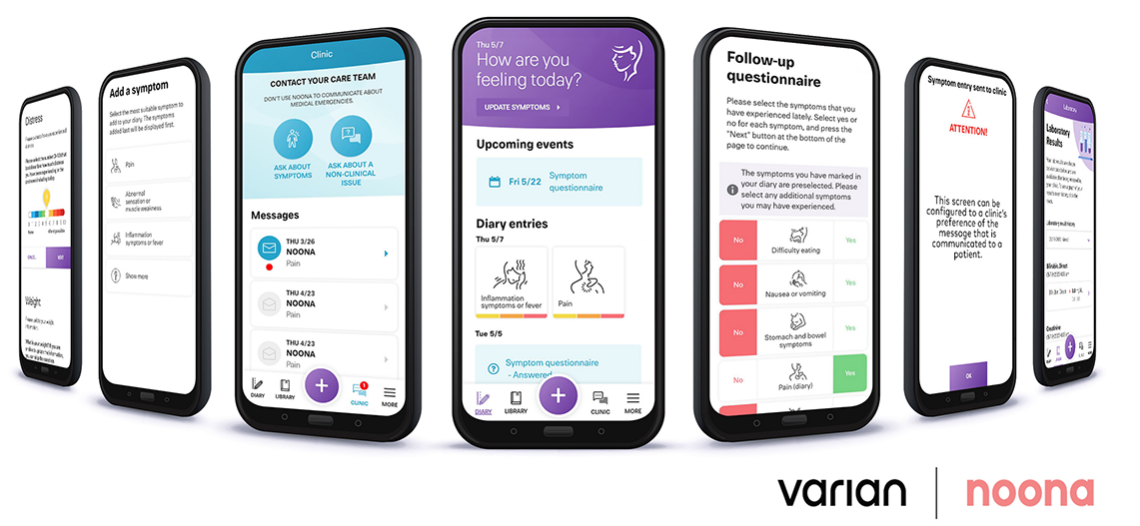
Noona interface for the patients.

**Figure 2:**
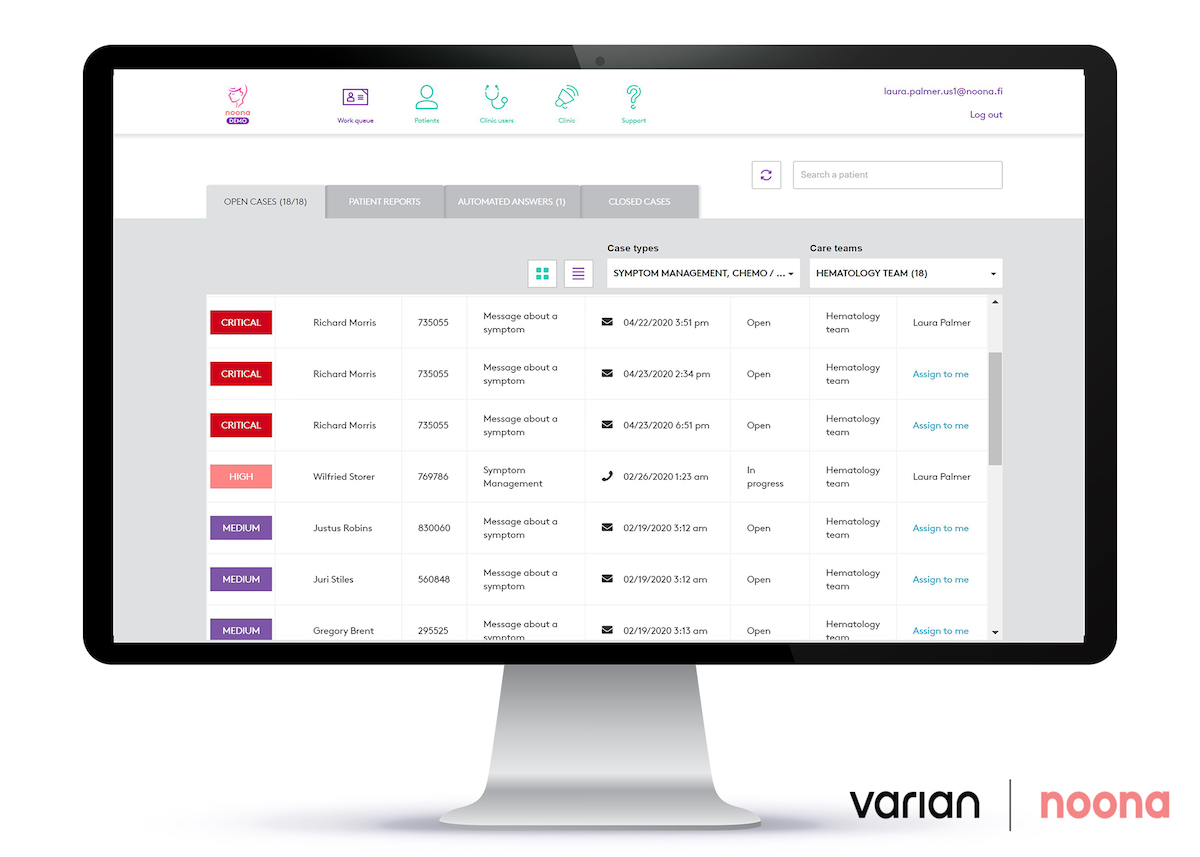
Noona interface for the professionals.

Noona has 2 user interfaces: one for the patients (Figure 1) and the other for the professionals (Figure 2). The main functionalities of the patient interface are symptom reporting and a diary. Patients can report on cancer- and treatment-related symptoms using question wizards that cover the clinically relevant questions and evaluate the most common and relevant symptoms, such as pain, fatigue, nausea, vomiting, and bowel symptoms. In addition to the symptoms, patients may contact their clinic regarding other topics using an open question form. Patients can also receive messages from their care team via Noona.

The main functionalities for the professionals are (1) a work queue to monitor new patients who have asked assistance or responded to a scheduled questionnaire, (2) a view of patient information as well as symptom history, and (3) a possibility to send messages directly to patients. When a patient starts to use Noona, the application provides a tutorial for the patient. As part of its implementation in the hospital, nurses are trained to give patients end user support. In the first version of Noona no alarm or reminder function was included and professionals were asked to check Noona regularly as part of their clinical work.

As part of the clinical evaluation of Noona, a prospective pilot trial was designed to study its usability and reliability by recruiting both patients with cancer and medical personnel, as both groups are expected to use the software during cancer treatments (ie, chemotherapy and radiotherapy or after cancer surgery). This clinical evaluation was part of the mandatory process before introducing Noona as a medical device to the markets in Finland and countries in Europe.

Patients were eligible if they were at least 18 years old; could read Finnish; received either radiotherapy, surgery, or medical therapy as a treatment for their cancer; and had a computer, phone, or tablet with an internet access available to be able to use Noona. Patients were asked to participate in the study by their physician during their routine visit at the hospital; otherwise their treatment continued according to local guidelines. All the doctors and nurses treating patients with cancer in the Helsinki University Comprehensive Cancer Center and willing to participate were eligible. Professionals (nurses, surgeons, medical oncologists, and radiotherapists) were recruited by the research doctor. A written informed consent was obtained from all participants. At the beginning, professionals had a training session about the use of Noona.

Patients were trained individually on the use of Noona by the research nurses and were introduced to the online tutorial. The information collected via Noona corresponded to that normally obtained in the clinical practice, and no additional information was requested. In case of a technical problem or if participants did not get a response from Noona within 24 hours, they were advised to contact their care team (nurse) by phone. Emergency patients were advised to contact their local emergency room.

A total of 45 patients with cancer and 18 health care professionals (nurses or medical doctors) were initially recruited at the Helsinki University Hospital Comprehensive Cancer Center in Finland in 2016. None of the participants declined to participate in the study; 1 patient and 1 professional were later excluded as they failed to fill in the questionnaire. Thus, the final number of patients and professionals recruited was 44 and 17, respectively. The study period was from June 26 to October 27, 2016, for the patients and from September 6 to November 1, 2016, for the professionals.

During the study period, the patients reported their symptoms and adverse events after surgery or during radiotherapy on Noona instead of reporting these via phone or at the doctor’s appointment. During chemotherapy the participating nurses sent their patients structured questionnaires via Noona a couple of days before chemotherapy infusion instead of contacting them over the phone. In addition, all patients could report using Noona how they are doing at any time. At the end of the study period, all participants answered questionnaires on the usability and reliability of Noona (Multimedia Appendix 1). Outcome measures of usability were ease of use, operability, and learnability. Participants were also asked about reliability (subjective opinion about Noona), whether they would recommend Noona to others, and if there was any harmful event related to the use of Noona. The questionnaires are described in detail in Multimedia Appendix 1.

The study was approved by the Ethics Committee of the Helsinki University Hospital.

## Results

### Patients

The mean age of the patients (n=44) was 55 (range 28-79); 37/44 (84%) patients were female and 7/44 (16%) were male. The cancer types were breast 32/44 (73%), gynecological 1/44 (2%), melanoma 3/44 (7%), colorectal 3/44 (7%), and urological 3/44 (7%). The information on cancer type was missing from 2/44 (5%) patients. Patients were either operated (13/44, 30%), received chemotherapy (16/44, 36%), or radiotherapy (14/44, 32%). The information on treatment type was missing from one patient (2%).

The patients were asked about their overall activity in using internet, mobile services, and their experiences of using Noona. About 70% (30/44, 68%) of the patients reported using mobile or web services every day (eg, when banking, shopping, or using social media). During the study Noona was used 1-3 times per week by 15/44 patients (34%) and less than that by 29/44 (66%). The most popular devices for the use of Noona were computer (19/44 patients, 43%), smartphone (10/44 patients, 23%), and tablet (7/44 patients, 16%), whereas others used more than 1 of these mentioned devices.

The detailed information about patients’ feedback on the usability of Noona in general, sending messages, and reporting of symptoms functions; their preference; and recommendation is presented in Table 1. Nearly 93% (41/44) of patients reported that the program was easy or quite easy to use, 38/44 (86%) patients found Noona reliable, and 38/44 (86%) patients would recommend Noona to other patients or their colleagues. No harmful incidences caused by the use of Noona were reported by the patients.

**Table 1:** Assessment of Noona’s usability by patients and professionals.

Question and parameter			Patients (n=44), n (%)		Professionals (n=17), n (%)
**Logging was**					
	Easy	30 (68)		14 (82)	
	Not easy or difficult	12 (27)		3 (18)	
	Difficult	2 (5)		0 (0)	
	Not answered	0 (0)		0 (0)	
**Enough instructions/education for use**					
	Yes	37 (84)		16 (94)	
	No	6 (14)		1 (6)	
	Not answered	1 (2)		0 (0)	
**Use of** **Noona** **was**					
	Easy	29 (66)		5 (29)	
	Quite easy	12 (27)		10 (59)	
	Quite difficult	1 (2)		1 (6)	
	Difficult	0 (0)		0 (0)	
	Not answered	2 (5)		1 (6)	
**Found message function**					
	Easy	22 (50)		12 (71)	
	Difficult	0 (0)		2 (12)	
	Not used the function	22 (50)		2 (12)	
	Not answered	0 (0)		1 (6)	
**Found the side effect reporting function**					
	Easy	20 (45)		Not asked in this group	
	Not easy or difficult	3 (7)			
	Difficult	0 (0)			
	Not used the function	21 (48)			
**Preference for communication**					
	Noona	7 (16)		Not asked in this group	
	Telephone	3 (7)			
	No preference	12 (27)			
	Not answered	22 (50)			
**Noona** **was reliable**					
	Yes	38 (86)		11 (65)	
	No	2 (5)		4 (24)	
	Not answered	4 (9)		2 (12)	
**Would recommend Noona**					
	Yes	38 (86)		10 (59)	
	No	2 (5)		2 (12)	
	Not answered or no comment	4 (9)		5 (29)	

### Health Care Professionals

A total of 7 physicians and 10 nurses participated in the study. The mean age of the health care professionals (n=17) was 43 (range 28-58), and 14/17 (82%) of them were female, 1/17 (6%) was male, and 2/17 (12%) did not report their gender. The specialties of the doctors were surgery (2/7, 29%), medical oncology (3/7, 43%), and radiotherapy (2/7, 29%); 3/10 (30%) nurses worked in the department of surgery, 5/10 (50%) in the department of chemotherapy, and 2/10 (20%) in the department of radiotherapy. All health care professionals reported, in general, using mobile or web services every day (several times a day). During the study, 8/17 (47%) professionals used Noona daily, 5/17 (29%) 1-3 times per week, and 4/17 (24%) less than that. A total of 3/7 (43%) doctors used Noona once a week while 4/7 (57%) used less than that; 8/10 nurses (80%) used Noona daily and 2/10 (20%) 1-3 times per week. The detailed information on the professionals’ feedback related to the use of Noona in general, message functions, their preference, and recommendation of Noona is presented in Table 1. As much as 15/17 (88%) professionals reported that the program was easy or quite easy to use and 11/17 (65%) professionals found Noona reliable; 10/17 (59%) professionals would recommend Noona to other patients or colleagues. One professional reported a harmful event with the use of Noona.

Participants spontaneously reported via the ePRO system that Noona “was easy to use” and “system was safe.” Some participants (13 patients and 12 professionals) gave suggestions for improvements. Recommendations included adding an alarm function when a new message has arrived, adding capability for users to send pictures through the program, possibility for logging in automatically with a saved password, and having more options for the questions.

## Discussion

### Principal Findings

This pilot study suggests that an ePRO application called Noona is feasible and acceptable in clinical practice. Most patients and professionals found Noona easy to log in and easy or quite easy to use. None of the patients reported difficulty with using Noona. Most of both patients and professionals would recommend Noona to other patients or colleagues.

None of the patients and only 1 professional reported a harmful event related to the use of Noona. In that case, the patient (female) did not understand that her symptoms were related to the treatment of cancer and thus did not report them via Noona; however, as per the professional, a direct face-to-face contact would have probably clarified the nature of her symptoms. None of the participants declined to participate in the study and only 2 participants were excluded from the study as they did not fill in the questionnaire. The high compliance rate suggests that participants found Noona easy enough to use. Only 2 patients had initial technical problems logging in, but these were subsequently resolved. One explanation for these promising results is that Noona has been designed in collaboration with the Helsinki University Hospital Comprehensive Cancer Center and the needs and requirements of both patients and professionals were recognized while developing Noona. A possible limitation of the study is that most participants had good digital literacy, which can limit the expansion of the results to different countries and populations.

As much as 8/10 (80%) of the nurses used Noona daily while none of the doctors did. This finding also reflects the current clinical practice, where most of the communication happens between patients and nurses.

Our results are in line with previous studies utilizing electronic data-collection tools [1,6-11], but currently no data comparing Noona with the other tools in a randomized setting are available. In their study of patients with breast cancer, Abernethy et al [6] demonstrated a high level of patient compliance and satisfaction using a tablet-based data-collection system. According to the study authors, the ePRO helped patients to identify symptoms that deserve reporting to their cancer care provider.

Advances in information technology have enabled the utilization of many ePRO systems in cancer clinics [12]. ePRO collection provides a unique opportunity to monitor symptoms in real-time and provide clinical management during cancer care [9]. Incorporation of the ePRO tool in clinical practice thus generates the opportunity to collect patient data via a comprehensive system [6]. Some studies have shown that ePROs have a positive impact on patients’ satisfaction [4,13], whereas others have found that ePROs are both feasible and acceptable [5,14-16] in clinical practice because published data show that their use may improve patient’s quality of life and even prognosis [4]. Because of these advantages, there is a growing interest in the use of internet-based follow-up systems.

### Conclusion

Noona seems to be an easy-to-use and suitable tool to monitor patient-reported outcomes during cancer treatments. However, larger studies are needed to compare ePROs with traditional methods of contact with regard to patient preference, quality of life, resource utility, and costs.

## Appendix

Multimedia Appendix 1 Questionnaire on the usability and reliability of Noona.

## Abbreviations

ePRO: electronic patient-reported outcome
