# Supplementary material for: A Novel Digital Patient-Reported Outcome Platform (Noona) for Clinical Use in Patients With Cancer: Pilot Study Assessing Suitability
Source: JMIR Form Res. 2021 May 6;5(5):e16156. doi: 10.2196/16156 (PMC8138702; doi:10.2196/16156)
Supplement: Multimedia Appendix 1 [file formative_v5i5e16156_app1.docx]

Supplement Table 1

Feedback Form for Patients


Date

Age

Gender

The cancer for which you are being treated

Module

Questions:

1.1 How often do you use network or mobile services?

Several times a day

Daily

2-3 times a day

Weekly

Rarely

1.2 How frequently have you used Noona during the study?

Several times a day

Daily

2-3 times a day

Weekly

Rarely

1.3 Which device you have used Noona with?

Computer

Smartphone

Tablet

2.1 Logging in Noona was

Easy

Not easy, but not difficult

Difficult

2.2 Noona guided me to get started well enough

I agree

I do not agree

3.1 Have you answered side effect questions during the study?

Yes

No

3.2 Answering the side effect questions in your opinion was

Easy

Not easy, but not difficult

Difficult

3.3 Sending the side effect questions in your opinion was

Easy

Not easy, but not difficult

Difficult

3.4 Did you use Noona sending questions to the clinic?

Yes

No

3.5 In your opinion sending questions was

Easy

Difficult

3.6 Did you get an answer to the questions you submitted through Noona?

Yes

No

3.7 In your opinion receiving the answer was

Easy

Difficult

3.8 Did you feel sending the question to the clinic by using Noona a more appropriate way than asking a question on the phone?

Yes

No

Both methods work equally well

4.1 In your opinion using Noona was

Easy

Quite easy

Quite difficult

Difficult

4.2 In your opinion Noona worked

Reliably

Unreliably

4.2.1 If Noona has worked unreliably, please describe what the possible problems have been?

4.3 Has using Noona caused you any inconvenience related to the disease or its treatment?

Yes

No

4.3.1 If harm has been caused, please describe what kind

4.4. Would you recommend Noona to other patients?

Yes

No

No comments

4.5 What could be improved in Noona?

Feedback Form for Professionals

Date

Age

Gender

Professional group: doctor / nurse

Module

Questions:

- 1. How often do you use network or mobile services?

Several times a day

Daily

2-3 times a day

Weekly

Rarely

- 1. How frequently have you used Noona during the study

Several times a day

Daily

2-3 times a day

Weekly

Rarely

2.1 Was the training provided on using Noona adequate?

Yes

No

2.2 Logging in Noona was

Easy

Not easy, but not difficult

Difficult

2.3 Creating patient IDs is easy

I agree

I do not agree

I have not used that feature

2.4 Adding basic patient information is easy

I agree

I do not agree

I have not used that feature

2.5 Renewing a patient password is easy

I agree

I do not agree

I have not used that feature

3.1 Patient answers to side effect questions and a pre-sent summary of responses reduce my time to treat the patient

I agree

I do not agree

I can not answer

I have not used that feature

3.2 Patient answers to treatment side effect questions and a pre-sent summary of responses can, in my judgment, increase patient safety

I agree

I do not agree

I can not answer

I have not used that feature

3.3 Patient answers to treatment side effect questions are usually sufficient to assess the patient's condition between reception visits

I agree

I do not agree

I can not answer

I have not used that feature

3.4 Answering patient questions through Noona is easy

I agree

I do not agree

I have not used that feature

4.1 In your opinion using Noona was

Easy

Quite easy

Quite difficult

Difficult

4.2 In your opinion Noona works

Reliably

Unreliably

4.2.1 If Noona has worked unreliably, please describe what the possible problems have been?

4.3 Has using Noona caused patient any inconvience related to the disease or its treatments?

Yes

No

4.3.1 If harm has been caused, please describe what kind?

4.4 Would you recommend Noona to other colleagues?

Yes

No

No comments

4.5 What could be improved in Noona?
